# Dynamic lifetime risk prediction of Alzheimer's disease with longitudinal cognitive assessment measurements

**DOI:** 10.1002/alz.70055

**Published:** 2025-03-05

**Authors:** Huitong Ding, Zehao Ye, Aris Paschalidis, David A. Bennett, Rhoda Au, Honghuang Lin

**Affiliations:** ^1^ Department of Anatomy and Neurobiology Boston University Chobanian & Avedisian School of Medicine Boston Massachusetts USA; ^2^ The Framingham Heart Study Boston University Chobanian & Avedisian School of Medicine Boston Massachusetts USA; ^3^ Department of Medicine University of Massachusetts Chan Medical School Worcester Massachusetts USA; ^4^ Rush Alzheimer's Disease Center Rush University Medical Center Chicago Illinois USA; ^5^ Department of Epidemiology Boston University School of Public Health Boston Massachusetts USA; ^6^ Department of Medicine Boston University Chobanian & Avedisian School of Medicine Boston Massachusetts USA; ^7^ Department of Neurology Boston University Chobanian & Avedisian School of Medicine Boston Massachusetts USA; ^8^ Slone Epidemiology Center Boston University Chobanian & Avedisian School of Medicine Boston Massachusetts USA

**Keywords:** Alzheimer's disease, cognitive assessment, dynamic risk prediction

## Abstract

**INTRODUCTION:**

The progressive nature of Alzheimer's disease (AD) highlights the importance of predicting lifetime risk and updating assessments as new data emerge. This study aimed to develop a dynamic model using longitudinal cognitive assessments for updated risk predictions.

**METHODS:**

This study used data from the Religious Orders Study and the Rush Memory and Aging Project (ROSMAP) to develop a dynamic risk prediction model based on five cognitive domains, updated annually over 10 years.

**RESULTS:**

The lifetime prediction models based on 2384 participants showed improved area under the curve (AUC) over time, rising from 0.578 at baseline to 0.765 with 10 years of data. The models predicting AD onset before ages 85 and 90 showed superior performance, with AUCs increasing from 0.761 to 0.932 and 0.658 to 0.876, respectively.

**DISCUSSION:**

Incorporating longitudinal cognitive assessments improves AD risk prediction as more data become available. Future research should integrate diverse data types to further boost predictive accuracy.

**Highlights:**

- Developed a dynamic lifetime risk prediction model.
- The area under the curve (AUC) increased from 0.578 at baseline to 0.765 with 10 years of data.
- The models predicting pre‐85 and pre‐90 risks demonstrated superior performance.

## 1 BACKGROUND

Alzheimer's disease (AD) represents a significant global health challenge, which is further compounded by an aging population worldwide.^1^ The limited availability of effective treatments for AD emphasizes the urgent need for robust strategies focused on early detection and precise lifetime risk prediction, which are vital for both optimal clinical management and the development of targeted interventions to mitigate the disease's impact.^2^ Accurate and individualized risk prediction is fundamental to precision medicine, emphasizing the value of models based on cognitive assessments. These models are noninvasive, cost‐effective, and easily integrated into epidemiological studies and clinical practice.^3^, ^4^, ^5^ Most risk prediction models rely on single‐time measures of risk factors.^6^, ^7^, ^8^, ^9^ However, AD is a progressive neurodegenerative disorder characterized by a gradual decline in cognitive function, with cognitive tests inherently time dependent.^10^, ^11^ Therefore, single‐time models may not effectively capture the dynamic changes in risk factors over time, which is critical for adapting interventions as the disease progresses.

Investigating the patterns and dynamic trends of cognitive changes throughout the course of cognitive decline is essential for advancing both research and clinical practice.^12^ Exploiting all the measures collected for a given individual until the current time point and continuously updating the prediction with each new measure could lead to more precise predictions. This approach ensures that the predictions reflect the most current information, accounting for individual changes over time. As such, dynamic modeling can lead to more accurate and timely predictions, enhancing our ability to understand and intervene in the progression of AD.

Effectively utilizing longitudinal measurements in AD research presents significant challenges, primarily due to the temporal dispersion of data types and the inconsistent administration frequencies of cognitive assessments. Many cohort studies have been conducted over decades, and earlier technological constraints meant that several potentially significant biomarkers were not collected extensively until more recent examinations. Moreover, as technology advances, new measurements, such as digital neuropsychological measures,^13^ are continually identified and incorporated into these studies, introducing new data streams. Integrating this continually expanding array of information to ensure that each participant's comprehensive history is accounted for in disease prediction poses substantial analytical challenges. Another complication arises from the dynamic nature of risk factors throughout an individual's life. Developing a statistical model that can adaptively characterize these age‐varying effects and provide robust and flexible risk assessments is not only crucial but also complex. This requires sophisticated modeling techniques that can handle the intricacies of longitudinal data and the impact of fluctuating risk factors to enhance predictive accuracy and disease understanding.

The objective of this study is to develop a dynamic lifetime risk prediction model utilizing longitudinal cognitive assessment data. This model aims to leverage all available measures collected for each individual up to the current time and dynamically update the predictions with each new measure. This approach ensures that the model continuously integrates the latest information, providing an evolving and accurate assessment of lifetime risk based on comprehensive historical data.

## 2 METHODS

### 2.1 Study population

The Religious Orders Study (ROS) and Rush Memory and Aging Project (MAP), collectively known as ROSMAP, are ongoing longitudinal cohort studies initiated in 1994 and 1997, respectively.^14^ These studies focus on aging and AD, with ROS enrolling nuns, priests, and brothers from across the United States and MAP primarily involving participants from Cook and the Collar counties in northeastern, Illinois. Participants in ROSMAP enroll without known dementia and undergo comprehensive, uniform evaluations, including self‐reported medical histories, neurological examinations, and cognitive function tests conducted by trained professionals. The details of the study design and data collection scheme have been reported previously.^14^, ^15^, ^16^ The study includes a large common core of data at the item level collected by a single team with the same trainer and study coordinator facilitating the efficient merging of data. All the research investigations received approval from the institutional review boards of Rush University, Columbia University, and Partners Healthcare/Broad Institute. Prior to participation, all individuals provided informed consent and signed a repository consent. The ROSMAP dataset used in this study was accessed on February 25, 2024.

### 2.2 Ascertainment of AD

**FIGURE 1 alz70055-fig-0001:**
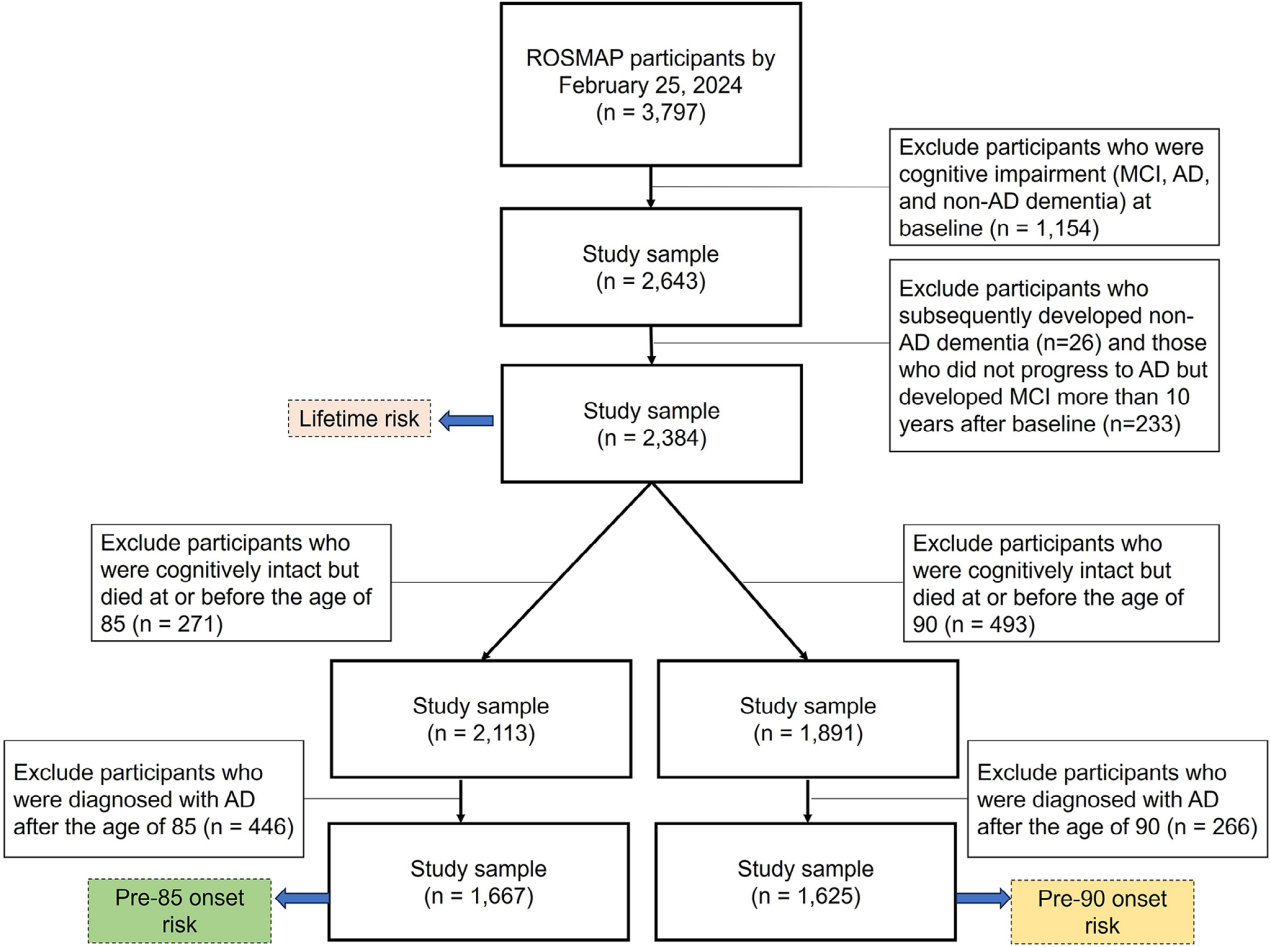
Sample selection diagram of this study.

The primary outcome of interest in this study was the lifetime risk of AD, defined as the probability of developing AD before death.^17^ Clinical diagnostic assessments occur at annual intervals and involve a combination of cognitive test scoring, clinical evaluations, and diagnostic classifications by clinicians, adhering to the criteria set by the National Institute of Neurological and Communicative Disorders and Stroke and the Alzheimer's Disease and Related Disorders Association (NINCDS‐ADRDA).^18^, ^19^ Diagnosing AD requires evidence of a substantial decline from previous cognitive performance, with deficits in memory and at least one additional cognitive domain. Participants with mild cognitive impairment (MCI) or dementia at baseline were excluded from this study (*n* = 1154). This study further excluded participants who subsequently developed non‐AD dementia (*n* = 26) and those who did not progress to AD but developed MCI more than 10 years after baseline (*n* = 233). Consequently, the analysis included 2384 participants who exhibited no cognitive impairment at baseline and had at least one clinical evaluation (Figure 1).

To address the limitations in prevalence and incidence estimates of AD in older age groups,^20^ we additionally performed a sensitivity analysis focusing on the risk of AD onset before the ages of 85 and 90 (Figure 1). In these analyses, we excluded participants who were cognitively intact but died at or before the age of 85 (*n* = 271) or 90 (*n* = 493), and we also excluded those diagnosed with AD after the age of 85 (*n* = 446) and 90 (*n* = 266) respectively. The final sample sizes for the analyses focusing on the risk of AD onset before the ages of 85 and 90 were 1667 and 1625 participants, respectively.

### 2.3 Neuropsychological assessment

For each participant in the study, comprehensive cognitive assessments were conducted at the initial assessment and during subsequent annual follow‐up visits. The methodology for neuropsychological (NP) assessment in ROSMAP and the computation of cognition domain scores has been detailed previously.^21^ We incorporated cognitive scores from five domains.^22^ Perceptual speed was assessed using four NP tests: Symbol Digits Modality Test (oral), Number Comparison, Stroop Color Naming, and Stroop Word Reading. Visuospatial ability was evaluated through two NP tests: Line Orientation and Progressive Matrices (16 items). Episodic memory was measured using seven NP tests, including Word List, Word List Recall, Word List Recognition, East Boston Immediate Recall, East Boston Delayed Recall, Logical Memory I (immediate recall), and Logical Memory II (delayed recall). Semantic memory was captured through three NP tests: Boston Naming (15 items), Category Fluency (Animals, Fruits/Vegetables), and Reading Test (10 items). Finally, working memory was assessed with three NP tests: Digits Forward, Digits Backward, and Digit Ordering. Each domain score was calculated by standardizing the raw scores of individual cognitive tests to z‐scores, based on the cohort's mean and standard deviation, and then computing the average of these z‐scores to obtain a composite scores.^22^ For participants diagnosed with incident AD, cognitive domain scores from all visits prior to the onset of AD were included in the analysis. For those without incident AD, cognitive assessments from all available visits were considered. In both cases, data were included for up to a decade, equating to a maximum of 11 visits per participant.

**RESEARCH IN CONTEXT**

1. **Systematic review**: We reviewed the literature using databases such as PubMed. Given the progressive nature of Alzheimer's disease (AD), static models cannot capture temporal changes in cognitive functions, underscoring the importance of utilizing temporal data to predict lifetime risk and dynamically update the risk assessment as new data become available.
2. **Interpretation**: Our study aimed to develop a dynamic lifetime risk prediction model capable of integrating longitudinal cognitive assessment measurements at time‐varying intervals to provide an updated probability of lifetime risk. Incorporating longitudinal cognitive assessments into risk prediction models enhances the ability to predict AD as more data become available.
3. **Future directions**: Future studies include exploring the integration of additional diverse data types to further enhance predictive performance.

### 2.4 Statistical analyses

To assess the demographic[Fig alz70055-fig-0001] and baseline characteristic differences between incident AD and cognitively intact groups, we applied the Kruskal‐Wallis H‐test for continuous variables and the chi‐square test for categorical variables. Significant differences were noted where the *p*‐values were less than 0.05.

**FIGURE 2:**
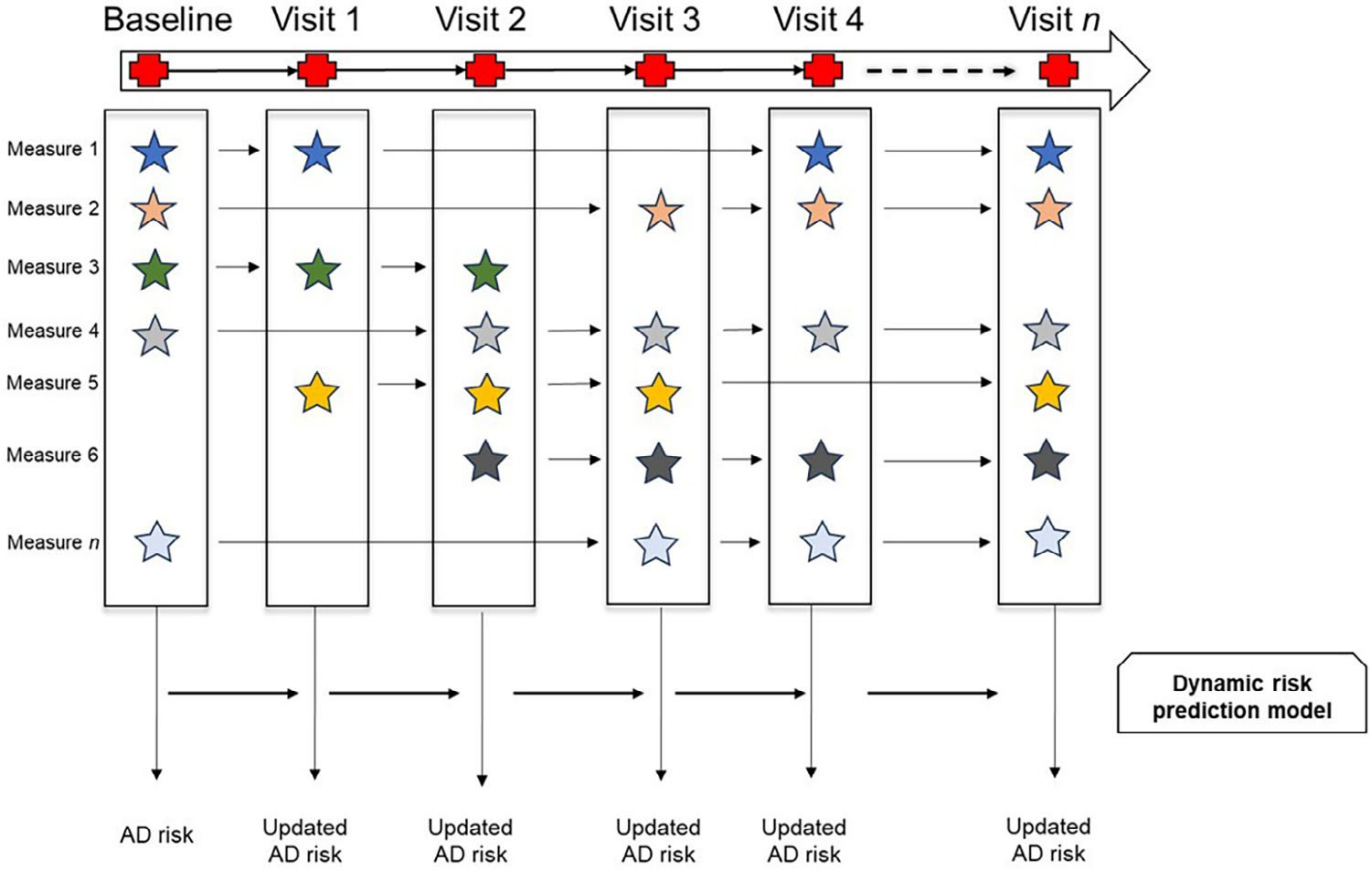
Dynamic risk prediction using multimodal measurements of a participant over time.

We constructed dynamic risk prediction models using data from five cognitive domains collected longitudinally over a period of up to 10 years to evaluate lifetime risk. Each model was developed using individual cognitive domain scores; in addition, a comprehensive model including all five cognitive domain scores as predictors was established. Each dynamic risk prediction model was constructed using a likelihood‐based two‐stage method (2SMLE) (Figure 2).^23^ In the first stage, the model captured the progression of cognitive decline over time through random effects generated by a linear mixed model, which is capable of handling missing values. ^24^ This model incorporated both random intercepts and slopes to address individual variations in baseline levels and their changes over time. Baseline age, sex, and education were included as covariates. This model's framework, employing restricted maximum likelihood for estimating fixed effects and the empirical best linear unbiased prediction for random effects, ensures that each participant's unique trajectory of cognitive changes is reflected accurately in the risk predictions. In the second stage, the point estimates of the participant‐specific random effects from the first stage, along with age, sex, and education level, serve as covariates in a logistic regression model to predict AD risk, which was fitted using the maximum likelihood estimation method.

The predictive performance of the model was evaluated using a bootstrap internal validation approach.^23^, ^25^ This approach employed the dynamic area under the receiver‐operating characteristic (ROC) curve (AUC(*t*)) as the primary discrimination metric for assessment. The AUC(*t*) was calculated from predictions based on longitudinal measurements gathered up to the specific time point *t*. To establish the reliability of this AUC(*t*) and to calculate its confidence intervals (CIs), we carried out a bootstrap procedure with 1000 iterations. In each iteration, a bootstrap sample was generated by randomly selecting patients with replacements from the original dataset. The DeLong test was used to examine if there was a significant difference between the two AUC values.^26^ Calibration was assessed using both Brier scores and calibration curves.^27^, ^28^ The calibration curve was generated by dividing participants into deciles of risk based on model predictions. For each decile, the observed incidence rate of AD was plotted against the average predicted probability of AD. The Brier score, a metric that evaluates the accuracy of probability predictions, ranges from 0 to 1, where lower scores indicate better alignment between predicted probabilities and actual outcomes. Further details on the evaluation methodology are available in previous studies.^23^, ^25^

We adopted an AUC‐based stepwise approach to identify the optimal combination of cognitive domains for predicting AD lifetime risk. Each cognitive domain was incorporated into individual models and assessed by bootstrapping AUC. Additional domains were subsequently added to the models one at a time. The final model included a combination of cognitive domains that reached the highest discriminative ability.

We also performed a sensitivity analysis to develop models based on three and four visits. For the three‐visit model, we selected the first visit, the last visit, and one randomly chosen intermediate visit for each participant. Similarly, for the four‐visit model, we included the first and last visits along with two randomly selected intermediate visits.

As an exploratory analysis, we developed models to evaluate the risk of AD onset occurring 5 and 10 years after the last included visit. For participants diagnosed with AD, all visits up to 5 or 10 years before their age of onset were included in the dataset for the 5‐year and 10‐year models. For participants without AD, only visits within 5 years prior to their last visit were included.

In addition, we developed dynamic prediction models to assess the risk of AD onset before the ages of 85 and 90, involving 1667 and 1625 participants, respectively, as depicted in Figure 1.

## 3 RESULTS

### 3.1 Cohort descriptive

This study involved 2384 participants for lifetime risk assessment (Table 1) and subsets of 1667 and 1625 participants for the pre‐85 and pre‐90 onset risks, respectively (Table S1). There were 617 cases of incident AD and 1318 deaths over an average of 8 years of follow‐up. Examination of specific age groups showed that the pre‐85 group experienced 171 cases of incident AD and 668 deaths over the 8‐year follow‐up, whereas the pre‐90 group had 351 cases of incident AD and 599 deaths. The average age of participants at baseline was in the mid‐70s across all groups and slightly older among the incident AD group (*p* < 0.001). A higher prevalence of women was noted for across all groups (77.3% and 77.4%, respectively). Education levels were high across the board, with most participants having received a college education or higher (64.0%). At baseline, participants demonstrated the following domain scores: perceptual speed (mean ± SD: 0.24 ± 0.76), visuospatial ability (mean 0.25 ± 0.69), episodic memory (mean 0.32 ± 0.48), semantic memory (mean 0.23 ± 0.58), and working memory (mean 0.17 ± 0.72). Higher scores in each domain reflect better cognitive performance. The average number of visits was 6.9 ± 3.1 for participants who were cognitively intact and 8.1 ± 2.8 for those with incident AD. Cognitive assessments at baseline revealed significant differences between the incident AD and cognitively intact groups; those with incident AD exhibited poorer performance in all five cognitive domains (*p* < 0.001).

**TABLE 1:** Baseline characteristics of participants for lifetime risk prediction.

Variable	Incident AD (*n* = 617)	Cognitively intact (*n* = 1767)	*p*
Age, years, mean ± SD	79 ± 7	77 ± 8	<0.001
Women, *n* (%)	477 (77.3)	1324 (74.9)	0.26
Education, *n* (%)			0.10
No high school	37 (6.0)	76 (4.3)	
High school	89 (14.4)	223 (12.6)	
Some college	96 (15.6)	328 (18.6)	
College and higher	395 (64.0)	1140 (64.5)	
Cognition, mean ± SD			
Perceptual speed	0.03 ± 0.77	0.31 ± 0.74	<0.001
Visuospatial ability	0.07 ± 0.69	0.32 ± 0.68	<0.001
Episodic memory	0.17 ± 0.47	0.37 ± 0.47	<0.001
Semantic memory	0.08 ± 0.59	0.28 ± 0.57	<0.001
Working memory	0.03 ± 0.71	0.22 ± 0.71	<0.001

Abbreviations: AD, Alzheimer's disease; SD, standard deviation.

### 3.2 Performance of dynamic prediction models for predicting lifetime risk

Table 2 illustrates the annual update of AUC for predicting AD lifetime risk using longitudinal measurements of five cognitive domains. Starting from a baseline AUC of 0.578 (95% CI: 0.547–0.608), the model exhibited a steady improvement in predictive performance, with the AUC rising to 0.765 (95% CI: 0.760–0.769) by Year 10.

**TABLE 2:** Annual update of AUC for predicting AD lifetime risk based on cognitive assessments over 10 years.

Year^a^	AUC	95% CI	
0	0.578	0.547	0.608
1	0.623	0.603	0.639
2	0.649	0.637	0.658
3	0.658	0.649	0.667
4	0.671	0.662	0.678
5	0.697	0.689	0.703
6	0.712	0.705	0.717
7	0.726	0.720	0.732
8	0.736	0.730	0.741
9	0.749	0.744	0.754
10	0.765	0.760	0.769

Abbreviations: AD, Alzheimer's disease; AUC, area under the curve; CI, confidence interval.

^a^ Year 0 represented the baseline. Each year following was labeled as year *n*, where “*n*” indicated the number of years post baseline. For each year *n*, the AUC was derived from a model that included data from the baseline year to the end of year *n*.

**FIGURE 3:**
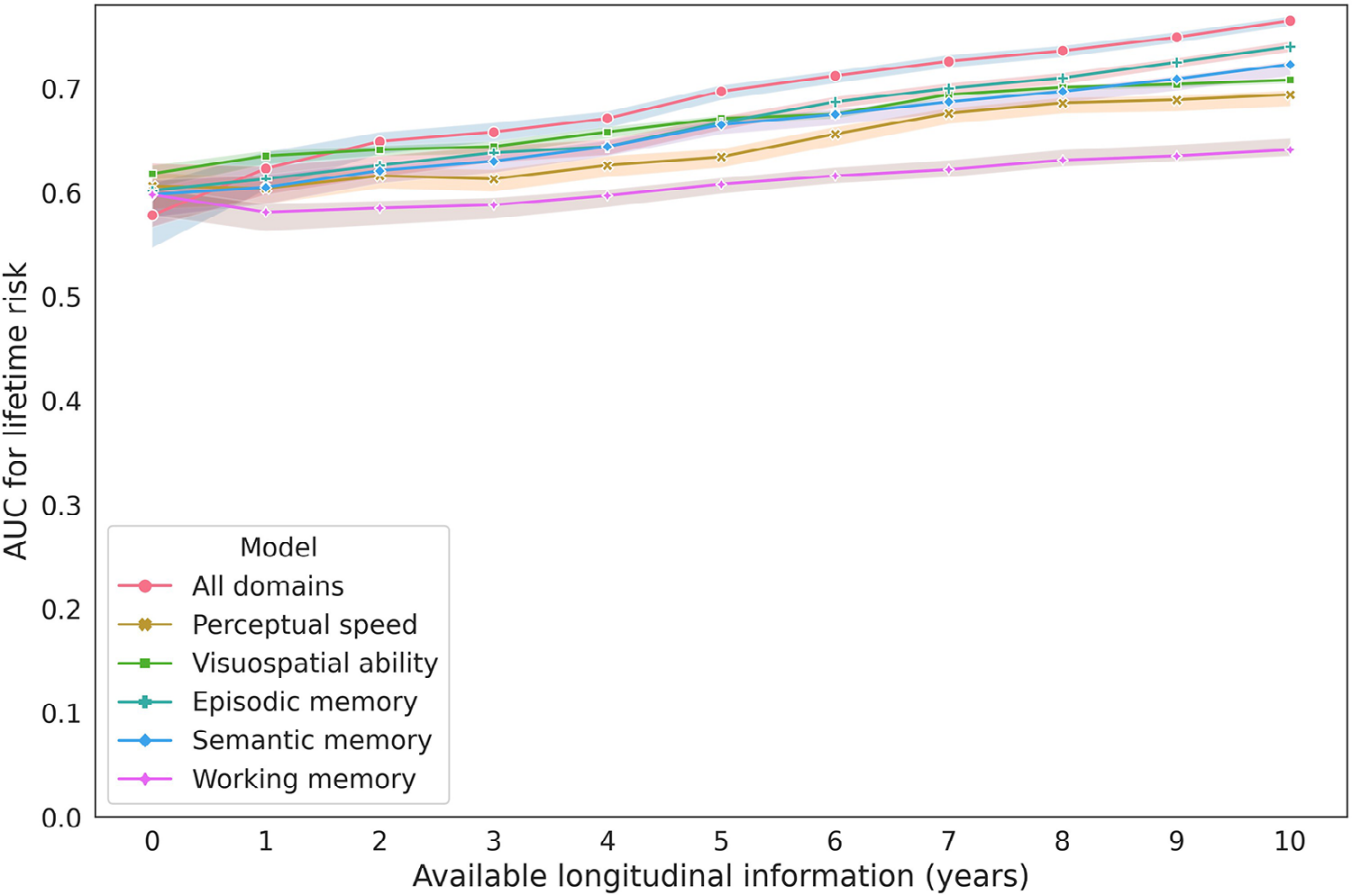
Updated AUC for lifetime risk of AD based on cognitive assessments over 10 years. Abbreviations: AD, Azheimer's disease; AUC, area under the curve.

We further examined the predictive performance based on individual cognitive domains. As shown in Table S2, the models based on cognitive function, controlling for demographics, generally showed a gradual improvement over time, with initial values around 0.598–0.618, increasing to 0.740 by Year 10 for the model based on episodic memory score, which, as expected, showed the best performance. The model based on five cognitive domains demonstrated superior performance compared to individual cognitive functions (Figure 3). Notably, starting from Year 8, the performance difference became statistically significant, with the model based on five cognitive domains showing a clear advantage over individual domain models (all *p*’s < 0.05). The model based on working memory consistently showed the lowest AUC values, although it does show a gradual increase. The predicted probabilities of AD risk aligned closely with the observed frequencies of AD risk, with a Brier score of 0.14 (Figure S1). The model's accurate calibration was further supported by a Brier score of 0.14. An illustrative example of two participants with different lifetime AD risk patterns is shown in Figure S2.

The stepwise approach revealed that incorporating three domains, including episodic memory, visuospatial ability, and semantic memory, achieved performance comparable to using all five domains (AUC 0.767) (Table S3). We further constructed separate lifetime risk prediction models for ROS and MAP. The results of these models consistently demonstrate improved predictive performance with the inclusion of longitudinal data. For ROS, the AUC increased from 0.591 at baseline to 0.753 with 10 years of data. Similarly, for MAP, the AUC rose from 0.616 at baseline to 0.796 with 10 years of data (Tables S4 and S5). The models based on the data of three and four visits shown consistently trend for performance improvement (Table S6). The exploratory analysis also revealed consistent performance trends for predicting 5‐year and 10‐year incident risk (Table S7).

### 3.3 Performance of dynamic prediction models for predicting pre‐85 and pre‐90 AD risk

We further assessed the AD risk before 85 and 90 years of age as shown in Tables S8–S11. Models that incorporated an increasing number of examinations consistently showed improved predictive performance. For the pre‐85 cohort, the cognitive model began with an AUC of 0.761 and steadily rose to 0.932 by Year 10. For models focusing on individual cognitive domains, episodic memory exhibited the highest AUC values, beginning at 0.737 and growing to as high as 0.900 by Year 10 (Table S9). Conversely, working memory displayed the lowest AUC, starting at 0.712 and modestly increasing to 0.778 by Year 10. Nearly all cognitive functions demonstrated a steady enhancement in their predictive capabilities, with annual increments in AUC.

The trend of incorporating an increasing number of examinations consistently showing improved predictive performance was similar for the pre‐90 AD risk prediction, starting with an AUC of 0.658 and rising to 0.876 with 10 years of data (Tables S10, S11). The model based on episodic memory continued to demonstrate the best predictive ability (AUC of 0.839 with 10 years of data), whereas the model based on working memory remained the weakest (AUC of 0.709 with 10 years of data). However, the predictive performance for the pre‐90 model was not as strong as that observed for the pre‐85 model.

## 4 DISCUSSION

In this study, we developed dynamic risk prediction models that utilize longitudinal cognitive measurements up to 10 years to evaluate the lifetime risk of AD, as well as the risk of AD onset before ages 85 and 90. These models are designed to accommodate data collected at varying intervals and are capable of dynamically updating predictions, as more data from participants become available. Our findings highlight that continual monitoring of cognitive functions and the integration of this information significantly improves the performance of lifetime risk predictions, starting with an AUC of 0.578 at baseline and progressively increasing to 0.765 with a full decade of data.

Predicting the lifetime risk of AD is crucial for facilitating early prevention strategies and optimizing care planning, enhancing the potential to delay onset and alter disease progression. Research has shown that delaying onset of AD symptoms by 5 years can reduce the risk of disease by as much as 50%.^29^ Traditional risk prediction models often rely on single‐time snapshots of risk factors. ^6^, ^7^, ^8^, ^9^ However, given the progressive nature of AD, these models may fall short because they do not reflect the ongoing changes in an individual's cognitive status. To overcome these limitations, our study tested a dynamic modeling approach that utilizes all available cognitive assessment data collected up to the current moment for each individual and updates predictions with each new data point. This approach ensures that predictions reflect the unique and evolving characteristics of each individual, capturing their dynamic progression over time. Our approach integrated longitudinal cognitive assessment measurements using summary measures that reflect their dynamic changes over time. These summary measures, in the form of latent variables such as subject‐specific random intercepts and slopes, harness the richness of longitudinal data within a mixed‐effects model framework. This methodology allows us to analyze individual differences in initial cognitive functions and their progression over time. It also accommodates various demographic factors that may influence the trajectory of clinical variables. Such a comprehensive method provides a nuanced understanding of the progression of cognitive decline in AD, making it possible to tailor interventions more precisely to the individual's changing risk profile. Moreover, our models are equipped to handle missing data effectively, ensuring robust predictions despite potential gaps in data collection or irregular intervals between assessments. This adaptability is vital for maintaining the accuracy and reliability of risk predictions in real‐world clinical and research settings, where data may not always be complete or uniformly collected. By dynamically integrating new information and accommodating missing data, we provide a more accurate and clinically useful tool for predicting the lifetime risk of AD, thereby supporting better clinical decision‐making and potentially improving outcomes for individuals at risk.

The findings of this study demonstrated improved performance over time, suggesting, not surprisingly, their growing reliability in AD risk prediction as more longitudinal data accumulates. For various cognitive subdomains, the predictive ability of episodic memory for lifetime, pre‐85, and pre‐90 AD onset risks was the highest. This finding aligns with previous research, as AD often begins with deficits in episodic memory.^30^ Conversely, the predictive ability of working memory for lifetime, pre‐85, and pre‐90 AD onset risks was the lowest. This may be because a decline in working memory is often associated with normal aging,^31^ making it challenging to distinguish from decline attributed specifically to AD. Unlike the pre‐85 and pre‐90 predictions where five cognitive domains consistently performed better than single domains, for the lifetime risk prediction, the combined cognitive domains did not perform as well as single domain at baseline. This discrepancy might be related to the pathogenesis of AD, where different cognitive functions decline at different times and rates. For example, deficits in episodic memory functioning are thought to occur early in the disease process, whereas visuospatial skills decline later.^32^, ^33^ In addition, in the sample, some incident AD cases might have late onset, thereby complicating baseline diagnoses. Therefore, at baseline, combining these cognitive domains might obscure the model's ability to distinguish between the different stages of cognitive decline. However, when multiple exam data points are available, the model can more accurately consider the trends in different cognitive functions. As a result, starting from measures after baseline, the performance of the model using all cognitive domains becomes optimal and maintains this improved performance thereafter. Furthermore, our study incorporates all available longitudinal information to continuously monitor cognitive health, although cognitive assessments closer to AD incidence are likely to better correlate with the clinical outcomes. This approach enables longitudinal assessment of AD risk across an individual's lifespan. Our results suggest that cognitive assessments provide a nuanced and sensitive approach to predicting AD risk. This could advocate for the integration of cognitive assessments into regular health evaluations for older adults to enhance early detection and potential mitigation of AD. Furthermore, the study revealed that models designed to predict AD onset before the ages of 85 and 90 yielded better performance than those estimating broader lifetime risks. This could be due to normal aging also causing cognitive decline, making it increasingly difficult to distinguish whether the decline is due to AD.^34^ In addition, this increased performance likely stems from these models' focus on a more specific and narrower age frame, which helps to minimize the impact of variability and potential confounding factors that are more prevalent in lifetime risk projections. By targeting this specific window, the models can provide more precise and actionable insights, potentially improving intervention strategies and outcomes for individuals at risk of developing AD at an earlier stage.

We acknowledge several limitations in our study. First, the older baseline age of our study population may constrain the wider applicability of our lifetime risk model, as it does not encompass younger age groups that might also benefit from early risk detection. To enhance the relevance and applicability of our findings, future studies should include a broader spectrum of age groups, thereby enriching the diversity of the data and extending the comprehensiveness of the lifetime risk predictions for AD. Our participants were predominantly non‐Latino White, and further work on more diverse cohorts is essential for generalization. In addition, this study did not account for the competing risk of death in relation to cognitive impairment. Despite the comprehensive approach to AD diagnosis, which incorporates selected information beyond cognitive assessments, we acknowledge the potential for a degree of circularity in our analysis. To mitigate this, we implemented measures to avoid bias by including only cognitive data from time points when participants were clinically classified as cognitively normal in the predictive modeling. This approach ensures that the data used reflect a cognitively healthy state, enabling the prediction of future AD risk without relying on data indicative of cognitive impairment. Furthermore, ROSMAP conducts annual assessments of participants’ cognitive status with an overall follow‐up rate over all years of more than 90%, offering a robust approximation of the age at onset of AD. This regular and systematic monitoring not only minimizes potential misclassification of cognitive states but also enhances the accuracy of early detection and risk prediction. Furthermore, this study focuses primarily on cognitive data, but future research will aim to expand the analysis by including other previously reported AD risk factors, such as blood pressure, glucose levels, body mass index, and lifestyle factors like physical activity, sleep, and diet that are often measured over time. The results presented here need to be externally validated with different cohorts to confirm their generalizability and robustness. This step is crucial to ensure that the dynamic risk prediction models we have developed perform consistently and effectively across varied demographic and clinical contexts, thus reinforcing their utility in real‐world settings. To include as many participants as possible while accommodating the constraints of limited follow‐up data, we opted for a binary outcome approach. Future studies could explore advanced predictive algorithms that leverage repeated measurements to better model and predict incident events.^35^

In summary, this study developed dynamic risk prediction models that leverage longitudinal cognitive measurements to assess lifetime AD risk. The incorporation of longitudinal data showed significant improvement in the prediction performance. Our study emphasizes the importance of continual cognitive monitoring and dynamic model updating to improve the early detection of AD.

## CONFLICT OF INTEREST STATEMENT

Dr. Au is a scientific advisor to Signant Health and NovoNordisk. The remaining authors declare that the research was conducted in the absence of any commercial or financial relationships that could be construed as a potential conflict of interest. Dr. Bennett reports no relevant conflicts of interest. Author disclosures are available in the supporting information.

## Supporting information



Supporting Information

Supporting Information

## References

[1] 2023 Alzheimer's disease facts and figures. Alzheimer Dement. 2023;19(4):1598‐1695. 10.1002/alz.13016 36918389

[2] Guo T , Zhang D , Zeng Y , Huang TY , Xu H , Zhao Y . Molecular and cellular mechanisms underlying the pathogenesis of Alzheimer's disease. Mol Neurodegen. 2020;15:1‐37. 10.1186/s13024-020-00391-7 PMC7364557 32677986

[3] Tang EY , Harrison SL , Errington L , et al. Current developments in dementia risk prediction modelling: an updated systematic review. PloS One. 2015;10(9):e0136181. 26334524 10.1371/journal.pone.0136181 PMC4559315

[4] Silva D , Guerreiro M , Santana I , et al. Prediction of long‐term (5 years) conversion to dementia using neuropsychological tests in a memory clinic setting. J Alzheimers Dis. 2013;34(3):681‐689. 23263232 10.3233/JAD-122098

[5] Jacqmin‐Gadda H , Blanche P , Chary E , Loubère L , Amieva H , Dartigues J‐F . Prognostic score for predicting risk of dementia over 10 years while accounting for competing risk of death. Am J Epidemiol. 2014;180(8):790‐798. 25190680 10.1093/aje/kwu202

[6] Ding H , Mandapati A , Hamel AP , et al. Multimodal machine learning for 10‐year dementia risk prediction: the Framingham Heart Study. J Alzheimer Dis. 2023:1‐10. (Preprint). 10.3233/JAD-230496 37742648

[7] Chary E , Amieva H , Pérès K , Orgogozo J‐M , Dartigues J‐F , Jacqmin‐Gadda H . Short‐versus long‐term prediction of dementia among subjects with low and high educational levels. Alzheimer Dement. 2013;9(5):562‐571. 10.1016/j.jalz.2012.05.2188 23159045

[8] Ding H , Mandapati A , Karjadi C , et al. Association between acoustic features and neuropsychological test performance in the Framingham Heart study: observational study. J Med Internet Res. 2022;24(12):e42886. 36548029 10.2196/42886 PMC9816957

[9] Ferretti MT , Ding H , Au R , et al. Maximizing utility of neuropsychological measures in sex‐specific predictive models of incident Alzheimer's disease in the Framingham Heart Study. Alzheimer Dement. 2024;20(2):1112‐1122. 10.1002/alz.13500 PMC10917035 37882354

[10] Morris JC . Mild cognitive impairment and preclinical Alzheimer's disease. Geriatrics. 2005(Suppl):9‐14. 16025770

[11] Ding H , Wang B , Hamel AP , et al. Exploring cognitive progression subtypes in the Framingham Heart Study. Alzheimer Dement. 2024;16(1):e12574. 10.1002/dad2.12574 PMC10955221 38515438

[12] Bettcher BM , Gross AL , Gavett BE , et al. Dynamic change of cognitive reserve: associations with changes in brain, cognition, and diagnosis. Neurobiol Aging. 2019;83:95‐104. 31585371 10.1016/j.neurobiolaging.2019.08.016 PMC6977973

[13] Au R , Popp ZT , Low S , et al. Digital is the new blood: enabling the present and the future. Alzheimer Dement. 2023;19:e078220.

[14] Bennett DA , Buchman AS , Boyle PA , Barnes LL , Wilson RS , Schneider JA . Religious orders study and rush memory and aging project. J Alzheimer Dis. 2018;64(s1):S161‐S189. 10.3233/JAD-179939 PMC6380522 29865057

[15] A Bennett D , A Schneider J , Arvanitakis Z , S Wilson R . Overview and findings from the religious orders study. Curr Alzheimer Res. 2012;9(6):628‐645. 22471860 10.2174/156720512801322573 PMC3409291

[16] A Bennett D , A Schneider J , S Buchman A , L Barnes L , A Boyle P , S Wilson R . Overview and findings from the rush Memory and Aging Project. Curr Alzheimer Res. 2012;9(6):646‐663. 22471867 10.2174/156720512801322663 PMC3439198

[17] Klijs B , Mitratza M , Harteloh PP , et al. Estimating the lifetime risk of dementia using nationwide individually linked cause‐of‐death and health register data. Int J Epidemiol. 2021;50(3):809‐816. 33354723 10.1093/ije/dyaa219

[18] Bennett DA , Schneider JA , Aggarwal NT , et al. Decision rules guiding the clinical diagnosis of Alzheimer's disease in two community‐based cohort studies compared to standard practice in a clinic‐based cohort study. Neuroepidemiology. 2006;27(3):169‐176. 17035694 10.1159/000096129

[19] McKhann G , Drachman D , Folstein M , Katzman R , Price D , Stadlan EM . Clinical diagnosis of Alzheimer's disease: report of the NINCDS‐ADRDA Work Group* under the auspices of Department of Health and Human Services Task Force on Alzheimer's Disease. Neurology. 1984;34(7):939‐939. 6610841 10.1212/wnl.34.7.939

[20] Kawas CH , Corrada MM . Alzheimer's and dementia in the oldest‐old: a century of challenges. Curr Alzheimer Res. 2006;3(5):411‐419. 17168640 10.2174/156720506779025233 PMC3373256

[21] Jacobs HI , Becker JA , Kwong K , et al. In vivo and neuropathology data support locus coeruleus integrity as indicator of Alzheimer's disease pathology and cognitive decline. Sci Trans Med. 2021;13(612):eabj2511. 10.1126/scitranslmed.abj2511 PMC8641759 34550726

[22] Wilson RS , Boyle PA , Yu L , et al. Temporal course and pathologic basis of unawareness of memory loss in dementia. Neurology. 2015;85(11):984‐991. 26311746 10.1212/WNL.0000000000001935 PMC4567465

[23] Dandis R , Teerenstra S , Massuger L , Sweep F , Eysbouts Y , IntHout J . A tutorial on dynamic risk prediction of a binary outcome based on a longitudinal biomarker. Biomet J. 2020;62(2):398‐413. 10.1002/bimj.201900044 PMC7079044 31777998

[24] Kumle L , Võ ML‐H , Draschkow D . Estimating power in (generalized) linear mixed models: an open introduction and tutorial in R. Behav Res Methods. 2021;53(6):2528‐2543. 33954914 10.3758/s13428-021-01546-0 PMC8613146

[25] Harrell Jr FE, Lee KL , Mark DB . Multivariable prognostic models: issues in developing models, evaluating assumptions and adequacy, and measuring and reducing errors. Stat Med. 1996;15(4):361‐387. 8668867 10.1002/(SICI)1097-0258(19960229)15:4<361::AID-SIM168>3.0.CO;2-4

[26] DeLong ER , DeLong DM , DL Clarke‐Pearson . Comparing the areas under two or more correlated receiver operating characteristic curves: a nonparametric approach. Biometrics. 1988:837‐845. 3203132

[27] Huang C‐C , Kuo W‐Y , Shen Y‐T , et al. Artificial intelligence prediction of in‐hospital mortality in patients with dementia: a multi‐center study. Int J Med Inform. 2024;191:105590. 39142178 10.1016/j.ijmedinf.2024.105590

[28] Le Scouarnec L , Bouteloup V , van der Veere PJ , et al. Development and assessment of algorithms for predicting brain amyloid positivity in a population without dementia. Res Ther. 2024;16(1):219. 10.1186/s13195-024-01595-5 PMC11468062 39394180

[29] Brookmeyer R , Gray S , Kawas C . Projections of Alzheimer's disease in the United States and the public health impact of delaying disease onset. Am J Public Health. 1998;88(9):1337‐1342. 9736873 10.2105/ajph.88.9.1337 PMC1509089

[30] Bäckman L , Small BJ , Fratiglioni L . Stability of the preclinical episodic memory deficit in Alzheimer's disease. Brain. 2001;124(1):96‐102. 11133790 10.1093/brain/124.1.96

[31] Tarawneh R , Holtzman DM . The clinical problem of symptomatic Alzheimer disease and mild cognitive impairment. Cold Spring Harbor Perspect Med. 2012;2(5):a006148. 10.1101/cshperspect.a006148 PMC3331682 22553492

[32] Small BJ , Herlitz A , Fratiglioni L , Almkvist O , Bäckman L . Cognitive predictors of incident Alzheimer's disease: a prospective longitudinal study. Neuropsychology. 1997;11(3):413. 9223145 10.1037//0894-4105.11.3.413

[33] Arriagada PV , Growdon JH , Hedley‐Whyte ET , Hyman BT . Neurofibrillary tangles but not senile plaques parallel duration and severity of Alzheimer's disease. Neurology. 1992;42(3):631‐631. 1549228 10.1212/wnl.42.3.631

[34] Murman DL . The Impact of Age on Cognition. Thieme Medical Publishers; 2015;36(3):111‐121. 10.1055/s-0035-1555115 PMC4906299 27516712

[35] Rizopoulos D . Dynamic predictions and prospective accuracy in joint models for longitudinal and time‐to‐event data. Biometrics. 2011;67(3):819‐829. 21306352 10.1111/j.1541-0420.2010.01546.x

