# Supplementary material for: Dynamic lifetime risk prediction of Alzheimer's disease with longitudinal cognitive assessment measurements
Source: Alzheimers Dement. 2025 Mar 5;21(3):e70055. doi: 10.1002/alz.70055 (PMC11881628; doi:10.1002/alz.70055)
Supplement: Supplementary file 2 — Supporting Information [file ALZ-21-e70055-s001.docx]

**Supplemental Table 1**. Baseline characteristics of participants for predicting AD onset before ages 85 and 90.

| **Variable** | **Pre-85 onset risk analysis** | | | **Pre-90 onset risk analysis** | | |
| --- | --- | --- | --- | --- | --- | --- |
|  | **Incident AD (n=171)** | **Cognitively intact (n=1496)** | ***P* value** | **Incident AD (n=351)** | **Cognitively intact (n=1274)** | ***P* value** |
| Age (years), mean±SD | 73±5 | 77±8 | <0.001 | 76±6 | 77±9 | 0.011 |
| Women, n (%) | 122(71.3) | 1156(77.3) | 0.10 | 263(74.9) | 1011(79.4) | 0.087 |
| Education, n (%) |  |  | 0.011 |  |  | 0.10 |
| No high school | 8(4.7) | 66(4.4) |  | 17(4.8) | 51(4.0) |  |
| High school | 23(13.5) | 172(11.5) |  | 50(14.2) | 150(11.8) |  |
| Some college | 15(8.8) | 286(19.1) |  | 48(13.7) | 241(18.9) |  |
| College and higher | 125(73.1) | 972(65.0) |  | 236(67.2) | 832(65.3) |  |
| **Cognition,** mean±SD |  |  |  |  |  |  |
| Perceptual speed | 0.01±0.76 | 0.33±0.73 | <0.001 | 0.04±0.76 | 0.37±0.73 | <0.001 |
| Visuospatial ability | 0.04±0.69 | 0.34±0.67 | <0.001 | 0.04±0.70 | 0.36±0.67 | <0.001 |
| Episodic memory | 0.19±0.48 | 0.38±0.48 | <0.001 | 0.15±0.48 | 0.40±0.48 | <0.001 |
| Semantic memory | 0.11±0.60 | 0.30±0.56 | <0.001 | 0.09±0.58 | 0.31±0.57 | <0.001 |
| Working memory | -0.05±0.77 | 0.24±0.71 | <0.001 | 0.04±0.73 | 0.25±0.71 | <0.001 |

**Supplemental Table 2.** The AUC for predicting AD lifetime risk based on individual cognitive domains over ten years.

| **Year*** | **Perceptual speed** | | | **Visuospatial ability** | | | **Episodic memory** | | | **Semantic memory** | | | **Working memory** | | |
| --- | --- | --- | --- | --- | --- | --- | --- | --- | --- | --- | --- | --- | --- | --- | --- |
|  | **AUC** | **95% CI** | | **AUC** | **95% CI** | | **AUC** | **95% CI** | | **AUC** | **95% CI** | | **AUC** | **95% CI** | |
| 0 | 0.606 | 0.584 | 0.619 | 0.618 | 0.604 | 0.626 | 0.602 | 0.567 | 0.628 | 0.598 | 0.576 | 0.612 | 0.598 | 0.578 | 0.604 |
| 1 | 0.604 | 0.589 | 0.614 | 0.635 | 0.627 | 0.640 | 0.613 | 0.598 | 0.625 | 0.605 | 0.589 | 0.617 | 0.581 | 0.563 | 0.589 |
| 2 | 0.616 | 0.604 | 0.624 | 0.641 | 0.635 | 0.645 | 0.626 | 0.615 | 0.635 | 0.621 | 0.609 | 0.629 | 0.585 | 0.569 | 0.592 |
| 3 | 0.613 | 0.601 | 0.622 | 0.644 | 0.637 | 0.649 | 0.638 | 0.629 | 0.645 | 0.630 | 0.619 | 0.637 | 0.588 | 0.575 | 0.595 |
| 4 | 0.626 | 0.615 | 0.635 | 0.658 | 0.652 | 0.663 | 0.644 | 0.636 | 0.651 | 0.644 | 0.635 | 0.650 | 0.597 | 0.586 | 0.603 |
| 5 | 0.634 | 0.624 | 0.642 | 0.671 | 0.666 | 0.674 | 0.667 | 0.660 | 0.673 | 0.665 | 0.656 | 0.670 | 0.608 | 0.599 | 0.614 |
| 6 | 0.656 | 0.645 | 0.663 | 0.675 | 0.671 | 0.678 | 0.687 | 0.681 | 0.692 | 0.675 | 0.665 | 0.680 | 0.616 | 0.609 | 0.624 |
| 7 | 0.676 | 0.666 | 0.681 | 0.694 | 0.691 | 0.696 | 0.700 | 0.694 | 0.705 | 0.687 | 0.676 | 0.691 | 0.622 | 0.615 | 0.631 |
| 8 | 0.686 | 0.676 | 0.691 | 0.701 | 0.698 | 0.703 | 0.710 | 0.704 | 0.715 | 0.697 | 0.686 | 0.700 | 0.631 | 0.625 | 0.641 |
| 9 | 0.689 | 0.678 | 0.693 | 0.704 | 0.701 | 0.705 | 0.725 | 0.720 | 0.729 | 0.709 | 0.698 | 0.712 | 0.635 | 0.630 | 0.646 |
| 10 | 0.694 | 0.683 | 0.698 | 0.708 | 0.705 | 0.709 | 0.740 | 0.735 | 0.745 | 0.723 | 0.712 | 0.726 | 0.641 | 0.635 | 0.652 |

*Year 0 represented the baseline. Each year following was labeled as year *n*, where '*n*' indicated the number of years post-baseline. For each year *n*, the AUC was derived from a model that included data from the baseline year to the end of year *n*. CI: confidence interval.

**Supplemental Table 3.** Optimal domain combinations identified through stepwise approach for AD lifetime risk prediction.

| **Year*** | **Two domains** | | | **Three domains** | | | **Four domains** | | |
| --- | --- | --- | --- | --- | --- | --- | --- | --- | --- |
|  | **Episodic memory +**  **visuospatial ability** | | | **Episodic memory +**  **visuospatial ability +**  **semantic memory** | | | **Episodic memory +**  **visuospatial ability +**  **semantic memory +**  **perceptual speed** | | |
|  | **AUC** | **95% CI** | | **AUC** | **95% CI** | | **AUC** | **95% CI** | |
| 0 | 0.600 | 0.574 | 0.622 | 0.592 | 0.557 | 0.616 | 0.579 | 0.553 | 0.607 |
| 1 | 0.634 | 0.621 | 0.643 | 0.634 | 0.613 | 0.644 | 0.626 | 0.609 | 0.640 |
| 2 | 0.650 | 0.641 | 0.658 | 0.652 | 0.640 | 0.659 | 0.650 | 0.640 | 0.659 |
| 3 | 0.660 | 0.652 | 0.667 | 0.662 | 0.651 | 0.668 | 0.660 | 0.651 | 0.667 |
| 4 | 0.672 | 0.665 | 0.679 | 0.674 | 0.664 | 0.679 | 0.673 | 0.664 | 0.679 |
| 5 | 0.697 | 0.691 | 0.701 | 0.701 | 0.694 | 0.705 | 0.699 | 0.691 | 0.704 |
| 6 | 0.714 | 0.709 | 0.717 | 0.715 | 0.709 | 0.719 | 0.714 | 0.708 | 0.719 |
| 7 | 0.729 | 0.725 | 0.732 | 0.729 | 0.723 | 0.733 | 0.730 | 0.723 | 0.733 |
| 8 | 0.738 | 0.733 | 0.740 | 0.738 | 0.732 | 0.742 | 0.739 | 0.732 | 0.742 |
| 9 | 0.750 | 0.745 | 0.753 | 0.752 | 0.747 | 0.755 | 0.752 | 0.747 | 0.755 |
| 10 | 0.764 | 0.760 | 0.766 | 0.767 | 0.763 | 0.769 | 0.767 | 0.762 | 0.770 |

*Year 0 represented the baseline. Each year following was labeled as year *n*, where '*n*' indicated the number of years post-baseline. For each year *n*, the AUC was derived from a model that included data from the baseline year to the end of year *n*. CI: confidence interval.

**Supplemental Table 4.** Annual update of AUC for predicting AD lifetime risk over ten years in ROS.

| **Year** | **All domains** | | | **Perceptual speed** | | | **Visuospatial ability** | | | **Episodic memory** | | | **Semantic memory** | | | **Working memory** | | |
| --- | --- | --- | --- | --- | --- | --- | --- | --- | --- | --- | --- | --- | --- | --- | --- | --- | --- | --- |
|  | **AUC** | **95% CI** | | **AUC** | **95% CI** | | **AUC** | **95% CI** | | **AUC** | **95% CI** | | **AUC** | **95% CI** | | **AUC** | **95% CI** | |
| 0 | 0.591 | 0.545 | 0.626 | 0.609 | 0.581 | 0.622 | 0.622 | 0.600 | 0.633 | 0.598 | 0.551 | 0.624 | 0.600 | 0.555 | 0.622 | 0.607 | 0.581 | 0.616 |
| 1 | 0.645 | 0.615 | 0.664 | 0.609 | 0.587 | 0.620 | 0.641 | 0.627 | 0.647 | 0.645 | 0.623 | 0.657 | 0.596 | 0.570 | 0.617 | 0.581 | 0.550 | 0.594 |
| 2 | 0.667 | 0.649 | 0.680 | 0.617 | 0.603 | 0.626 | 0.638 | 0.625 | 0.646 | 0.657 | 0.643 | 0.667 | 0.622 | 0.608 | 0.634 | 0.575 | 0.551 | 0.588 |
| 3 | 0.699 | 0.685 | 0.709 | 0.621 | 0.609 | 0.630 | 0.647 | 0.636 | 0.654 | 0.693 | 0.685 | 0.700 | 0.640 | 0.627 | 0.649 | 0.587 | 0.571 | 0.598 |
| 4 | 0.693 | 0.679 | 0.703 | 0.633 | 0.621 | 0.641 | 0.659 | 0.649 | 0.665 | 0.686 | 0.676 | 0.694 | 0.637 | 0.626 | 0.646 | 0.606 | 0.594 | 0.613 |
| 5 | 0.709 | 0.698 | 0.716 | 0.645 | 0.635 | 0.651 | 0.664 | 0.655 | 0.670 | 0.698 | 0.690 | 0.704 | 0.663 | 0.652 | 0.671 | 0.618 | 0.608 | 0.625 |
| 6 | 0.712 | 0.701 | 0.720 | 0.662 | 0.653 | 0.668 | 0.668 | 0.660 | 0.675 | 0.702 | 0.693 | 0.709 | 0.664 | 0.654 | 0.672 | 0.628 | 0.620 | 0.636 |
| 7 | 0.718 | 0.707 | 0.727 | 0.674 | 0.667 | 0.679 | 0.678 | 0.671 | 0.685 | 0.709 | 0.700 | 0.716 | 0.673 | 0.663 | 0.681 | 0.632 | 0.622 | 0.640 |
| 8 | 0.722 | 0.711 | 0.731 | 0.682 | 0.674 | 0.686 | 0.684 | 0.677 | 0.691 | 0.714 | 0.704 | 0.721 | 0.682 | 0.672 | 0.690 | 0.634 | 0.625 | 0.643 |
| 9 | 0.734 | 0.724 | 0.741 | 0.676 | 0.668 | 0.682 | 0.686 | 0.678 | 0.693 | 0.727 | 0.717 | 0.734 | 0.695 | 0.686 | 0.704 | 0.640 | 0.632 | 0.651 |
| 10 | 0.753 | 0.744 | 0.760 | 0.678 | 0.669 | 0.684 | 0.688 | 0.681 | 0.697 | 0.744 | 0.735 | 0.750 | 0.710 | 0.701 | 0.720 | 0.650 | 0.641 | 0.663 |

*Year 0 represented the baseline. Each year following was labeled as year *n*, where '*n*' indicated the number of years post-baseline. For each year *n*, the AUC was derived from a model that included data from the baseline year to the end of year *n*. CI: confidence interval.

**Supplemental Table 5.** Annual update of AUC for predicting AD lifetime risk over ten years in MAP.

| **Year** | **All domains** | | | **Perceptual speed** | | | **Visuospatial ability** | | | **Episodic memory** | | | **Semantic memory** | | | **Working memory** | | |
| --- | --- | --- | --- | --- | --- | --- | --- | --- | --- | --- | --- | --- | --- | --- | --- | --- | --- | --- |
|  | **AUC** | **95% CI** | | **AUC** | **95% CI** | | **AUC** | **95% CI** | | **AUC** | **95% CI** | | **AUC** | **95% CI** | | **AUC** | **95% CI** | |
| 0 | 0.616 | 0.551 | 0.660 | 0.644 | 0.625 | 0.653 | 0.632 | 0.617 | 0.640 | 0.665 | 0.635 | 0.679 | 0.636 | 0.615 | 0.643 | 0.635 | 0.620 | 0.641 |
| 1 | 0.630 | 0.598 | 0.654 | 0.637 | 0.620 | 0.647 | 0.637 | 0.626 | 0.644 | 0.639 | 0.618 | 0.655 | 0.638 | 0.621 | 0.647 | 0.627 | 0.614 | 0.633 |
| 2 | 0.654 | 0.635 | 0.669 | 0.653 | 0.638 | 0.663 | 0.647 | 0.637 | 0.653 | 0.655 | 0.639 | 0.669 | 0.636 | 0.620 | 0.648 | 0.639 | 0.628 | 0.645 |
| 3 | 0.648 | 0.632 | 0.662 | 0.646 | 0.631 | 0.657 | 0.647 | 0.635 | 0.654 | 0.643 | 0.627 | 0.657 | 0.644 | 0.630 | 0.654 | 0.638 | 0.627 | 0.644 |
| 4 | 0.676 | 0.663 | 0.687 | 0.663 | 0.649 | 0.673 | 0.662 | 0.652 | 0.669 | 0.665 | 0.651 | 0.677 | 0.675 | 0.664 | 0.684 | 0.636 | 0.624 | 0.644 |
| 5 | 0.706 | 0.694 | 0.716 | 0.663 | 0.650 | 0.674 | 0.682 | 0.674 | 0.687 | 0.693 | 0.682 | 0.703 | 0.695 | 0.684 | 0.702 | 0.645 | 0.635 | 0.652 |
| 6 | 0.729 | 0.718 | 0.738 | 0.688 | 0.676 | 0.696 | 0.687 | 0.680 | 0.692 | 0.721 | 0.711 | 0.728 | 0.707 | 0.696 | 0.714 | 0.651 | 0.641 | 0.660 |
| 7 | 0.753 | 0.742 | 0.762 | 0.711 | 0.701 | 0.717 | 0.713 | 0.705 | 0.717 | 0.737 | 0.729 | 0.744 | 0.719 | 0.709 | 0.727 | 0.661 | 0.650 | 0.669 |
| 8 | 0.768 | 0.758 | 0.776 | 0.725 | 0.716 | 0.729 | 0.721 | 0.713 | 0.725 | 0.751 | 0.744 | 0.758 | 0.730 | 0.720 | 0.737 | 0.678 | 0.669 | 0.687 |
| 9 | 0.784 | 0.775 | 0.791 | 0.732 | 0.723 | 0.736 | 0.725 | 0.716 | 0.729 | 0.768 | 0.761 | 0.775 | 0.741 | 0.731 | 0.747 | 0.685 | 0.675 | 0.693 |
| 10 | 0.796 | 0.787 | 0.803 | 0.739 | 0.729 | 0.742 | 0.731 | 0.721 | 0.735 | 0.778 | 0.770 | 0.785 | 0.753 | 0.743 | 0.758 | 0.686 | 0.675 | 0.694 |

*Year 0 represented the baseline. Each year following was labeled as year *n*, where '*n*' indicated the number of years post-baseline. For each year *n*, the AUC was derived from a model that included data from the baseline year to the end of year *n*. CI: confidence interval.

**Supplemental Table 6.** Update of AUC for predicting AD lifetime risk based on three and four visit data.

| **Visit** | **Three-visit model** | | | **Four-visit model** | | |
| --- | --- | --- | --- | --- | --- | --- |
|  | **AUC** | **95% CI** | | **AUC** | **95% CI** | |
| 1 | 0.598 | 0.564 | 0.623 | 0.591 | 0.562 | 0.623 |
| 2 | 0.655 | 0.647 | 0.664 | 0.673 | 0.663 | 0.683 |
| 3 | 0.789 | 0.785 | 0.791 | 0.691 | 0.684 | 0.699 |
| 4 | - | - | - | 0.785 | 0.783 | 0.789 |

**Supplemental Table 7.** Annual update of AUC for predicting incident AD risk based on 5 cognitive domains for the next 5 and 10 years.

| **Year*** | **5-year** | | | **10-year** | | |
| --- | --- | --- | --- | --- | --- | --- |
|  | **AUC** | **95% CI** | | **AUC** | **95% CI** | |
| 0 | 0.678 | 0.661 | 0.685 | 0.713 | 0.692 | 0.723 |
| 1 | 0.689 | 0.681 | 0.694 | 0.717 | 0.697 | 0.724 |
| 2 | 0.697 | 0.689 | 0.702 | 0.716 | 0.700 | 0.723 |
| 3 | 0.709 | 0.700 | 0.714 | 0.729 | 0.717 | 0.736 |
| 4 | 0.714 | 0.706 | 0.721 | 0.746 | 0.737 | 0.751 |
| 5 | 0.730 | 0.722 | 0.737 | 0.755 | 0.747 | 0.761 |
| 6 | 0.743 | 0.736 | 0.750 | 0.762 | 0.754 | 0.769 |
| 7 | 0.755 | 0.748 | 0.764 | 0.769 | 0.761 | 0.777 |
| 8 | 0.764 | 0.757 | 0.772 | 0.776 | 0.768 | 0.785 |
| 9 | 0.773 | 0.765 | 0.780 | 0.781 | 0.774 | 0.789 |
| 10 | 0.782 | 0.775 | 0.790 | 0.785 | 0.779 | 0.794 |

*Year 0 represented the baseline. Each year following was labeled as year *n*, where '*n*' indicated the number of years post-baseline. For each year *n*, the AUC was derived from a model that included data from the baseline year to the end of year *n*. CI: confidence interval.

**Supplemental Table 8.** Annual update of AUC for predicting incident AD risk before age 85 based on 5 cognitive domains over ten years.

| **Year*** | **AUC** | **95% CI** | |
| --- | --- | --- | --- |
| 0 | 0.761 | 0.742 | 0.777 |
| 1 | 0.797 | 0.785 | 0.807 |
| 2 | 0.828 | 0.817 | 0.834 |
| 3 | 0.851 | 0.841 | 0.860 |
| 4 | 0.873 | 0.861 | 0.882 |
| 5 | 0.888 | 0.880 | 0.895 |
| 6 | 0.904 | 0.896 | 0.910 |
| 7 | 0.913 | 0.907 | 0.918 |
| 8 | 0.916 | 0.910 | 0.921 |
| 9 | 0.923 | 0.916 | 0.927 |
| 10 | 0.932 | 0.926 | 0.935 |

*Year 0 represented the baseline. Each year following was labeled as year *n*, where '*n*' indicated the number of years post-baseline. For each year *n*, the AUC was derived from a model that included data from the baseline year to the end of year *n*. CI: confidence interval.

**Supplemental table 9.** Annual update of AUC for predicting incident AD risk before age 85 based on individual cognitive domains over ten years.

| **Year*** | **Perceptual speed** | | | **Visuospatial ability** | | | **Episodic memory** | | | **Semantic memory** | | | **Working memory** | | |
| --- | --- | --- | --- | --- | --- | --- | --- | --- | --- | --- | --- | --- | --- | --- | --- |
|  | **AUC** | **95% CI** | | **AUC** | **95% CI** | | **AUC** | **95% CI** | | **AUC** | **95% CI** | | **AUC** | **95% CI** | |
| 0 | 0.755 | 0.744 | 0.762 | 0.733 | 0.724 | 0.739 | 0.737 | 0.715 | 0.744 | 0.715 | 0.698 | 0.725 | 0.712 | 0.700 | 0.719 |
| 1 | 0.759 | 0.749 | 0.766 | 0.766 | 0.757 | 0.770 | 0.761 | 0.752 | 0.767 | 0.730 | 0.716 | 0.738 | 0.713 | 0.700 | 0.723 |
| 2 | 0.782 | 0.772 | 0.788 | 0.781 | 0.775 | 0.785 | 0.788 | 0.777 | 0.794 | 0.763 | 0.748 | 0.770 | 0.725 | 0.712 | 0.733 |
| 3 | 0.800 | 0.790 | 0.807 | 0.793 | 0.787 | 0.797 | 0.808 | 0.798 | 0.815 | 0.801 | 0.788 | 0.808 | 0.714 | 0.700 | 0.725 |
| 4 | 0.810 | 0.799 | 0.818 | 0.809 | 0.803 | 0.814 | 0.824 | 0.815 | 0.831 | 0.823 | 0.811 | 0.831 | 0.738 | 0.727 | 0.746 |
| 5 | 0.822 | 0.811 | 0.830 | 0.818 | 0.812 | 0.823 | 0.847 | 0.839 | 0.852 | 0.842 | 0.831 | 0.850 | 0.742 | 0.731 | 0.752 |
| 6 | 0.843 | 0.833 | 0.850 | 0.824 | 0.819 | 0.829 | 0.867 | 0.861 | 0.872 | 0.849 | 0.838 | 0.856 | 0.758 | 0.748 | 0.766 |
| 7 | 0.858 | 0.849 | 0.864 | 0.841 | 0.835 | 0.845 | 0.874 | 0.868 | 0.878 | 0.861 | 0.851 | 0.867 | 0.764 | 0.753 | 0.771 |
| 8 | 0.862 | 0.853 | 0.868 | 0.844 | 0.838 | 0.847 | 0.882 | 0.876 | 0.886 | 0.868 | 0.859 | 0.873 | 0.764 | 0.753 | 0.771 |
| 9 | 0.870 | 0.861 | 0.875 | 0.845 | 0.839 | 0.849 | 0.891 | 0.886 | 0.894 | 0.873 | 0.864 | 0.878 | 0.772 | 0.761 | 0.779 |
| 10 | 0.873 | 0.864 | 0.879 | 0.851 | 0.844 | 0.855 | 0.900 | 0.894 | 0.903 | 0.882 | 0.873 | 0.886 | 0.778 | 0.768 | 0.785 |

*Year 0 represented the baseline. Each year following was labeled as year *n*, where '*n*' indicated the number of years post-baseline. For each year *n*, the AUC was derived from a model that included data from the baseline year to the end of year *n*. CI: confidence interval.

**Supplemental Table 10.** Annual update of AUC for predicting incident AD risk before age 90 based on 5 cognitive domains over ten years.

| **Year*** | **AUC** | **95% CI** | |
| --- | --- | --- | --- |
| 0 | 0.658 | 0.638 | 0.687 |
| 1 | 0.717 | 0.701 | 0.734 |
| 2 | 0.754 | 0.744 | 0.764 |
| 3 | 0.783 | 0.776 | 0.791 |
| 4 | 0.805 | 0.799 | 0.813 |
| 5 | 0.824 | 0.817 | 0.830 |
| 6 | 0.840 | 0.834 | 0.846 |
| 7 | 0.850 | 0.844 | 0.855 |
| 8 | 0.855 | 0.849 | 0.860 |
| 9 | 0.866 | 0.861 | 0.870 |
| 10 | 0.876 | 0.872 | 0.880 |

*Year 0 represented the baseline. Each year following was labeled as year *n*, where '*n*' indicated the number of years post-baseline. For each year *n*, the AUC was derived from a model that included data from the baseline year to the end of year *n*. CI: confidence interval.

**Supplemental table 11.** Annual update of AUC for predicting incident AD risk before age 90 based on individual cognitive domains over ten years.

| **Year*** | **Perceptual speed** | | | **Visuospatial ability** | | | **Episodic memory** | | | **Semantic memory** | | | **Working memory** | | |
| --- | --- | --- | --- | --- | --- | --- | --- | --- | --- | --- | --- | --- | --- | --- | --- |
|  | **AUC** | **95% CI** | | **AUC** | **95% CI** | | **AUC** | **95% CI** | | **AUC** | **95% CI** | | **AUC** | **95% CI** | |
| 0 | 0.660 | 0.643 | 0.667 | 0.650 | 0.641 | 0.657 | 0.649 | 0.626 | 0.675 | 0.628 | 0.604 | 0.648 | 0.601 | 0.575 | 0.607 |
| 1 | 0.676 | 0.664 | 0.682 | 0.681 | 0.675 | 0.685 | 0.692 | 0.679 | 0.704 | 0.655 | 0.640 | 0.667 | 0.593 | 0.576 | 0.600 |
| 2 | 0.704 | 0.694 | 0.710 | 0.700 | 0.695 | 0.704 | 0.717 | 0.706 | 0.726 | 0.685 | 0.672 | 0.695 | 0.612 | 0.599 | 0.618 |
| 3 | 0.727 | 0.719 | 0.733 | 0.714 | 0.709 | 0.718 | 0.744 | 0.735 | 0.751 | 0.716 | 0.705 | 0.724 | 0.629 | 0.619 | 0.634 |
| 4 | 0.741 | 0.734 | 0.746 | 0.732 | 0.727 | 0.736 | 0.755 | 0.746 | 0.761 | 0.739 | 0.730 | 0.746 | 0.648 | 0.640 | 0.653 |
| 5 | 0.755 | 0.748 | 0.760 | 0.745 | 0.741 | 0.749 | 0.781 | 0.775 | 0.787 | 0.757 | 0.748 | 0.763 | 0.661 | 0.651 | 0.666 |
| 6 | 0.774 | 0.768 | 0.779 | 0.758 | 0.753 | 0.761 | 0.803 | 0.796 | 0.808 | 0.776 | 0.767 | 0.782 | 0.680 | 0.672 | 0.685 |
| 7 | 0.790 | 0.785 | 0.795 | 0.775 | 0.771 | 0.778 | 0.809 | 0.803 | 0.814 | 0.784 | 0.776 | 0.790 | 0.688 | 0.680 | 0.694 |
| 8 | 0.797 | 0.792 | 0.802 | 0.784 | 0.780 | 0.787 | 0.815 | 0.808 | 0.820 | 0.793 | 0.784 | 0.798 | 0.695 | 0.687 | 0.700 |
| 9 | 0.801 | 0.796 | 0.806 | 0.790 | 0.786 | 0.794 | 0.829 | 0.823 | 0.834 | 0.801 | 0.793 | 0.806 | 0.702 | 0.694 | 0.708 |
| 10 | 0.805 | 0.800 | 0.810 | 0.795 | 0.790 | 0.800 | 0.839 | 0.835 | 0.844 | 0.814 | 0.805 | 0.818 | 0.709 | 0.701 | 0.715 |

*Year 0 represented the baseline. Each year following was labeled as year *n*, where '*n*' indicated the number of years post-baseline. For each year *n*, the AUC was derived from a model that included data from the baseline year to the end of year *n*. CI: confidence interval.

**Supplemental figure 1**. Calibration plot of predicted probability against observed frequency of AD. The x-axis represents the probability of AD as estimated by the prediction model based on 10-year data. Participants were grouped into probability intervals (e.g., 0.0–0.1, 0.1–0.2, etc.), and each point reflects the average probability for participants within a specific group. The y-axis shows the actual percentage of participants diagnosed with AD, where each point indicates the observed percentage within a probability group. The dashed line represents a perfect match between the predicted probabilities and the observed diagnosis rates.

**
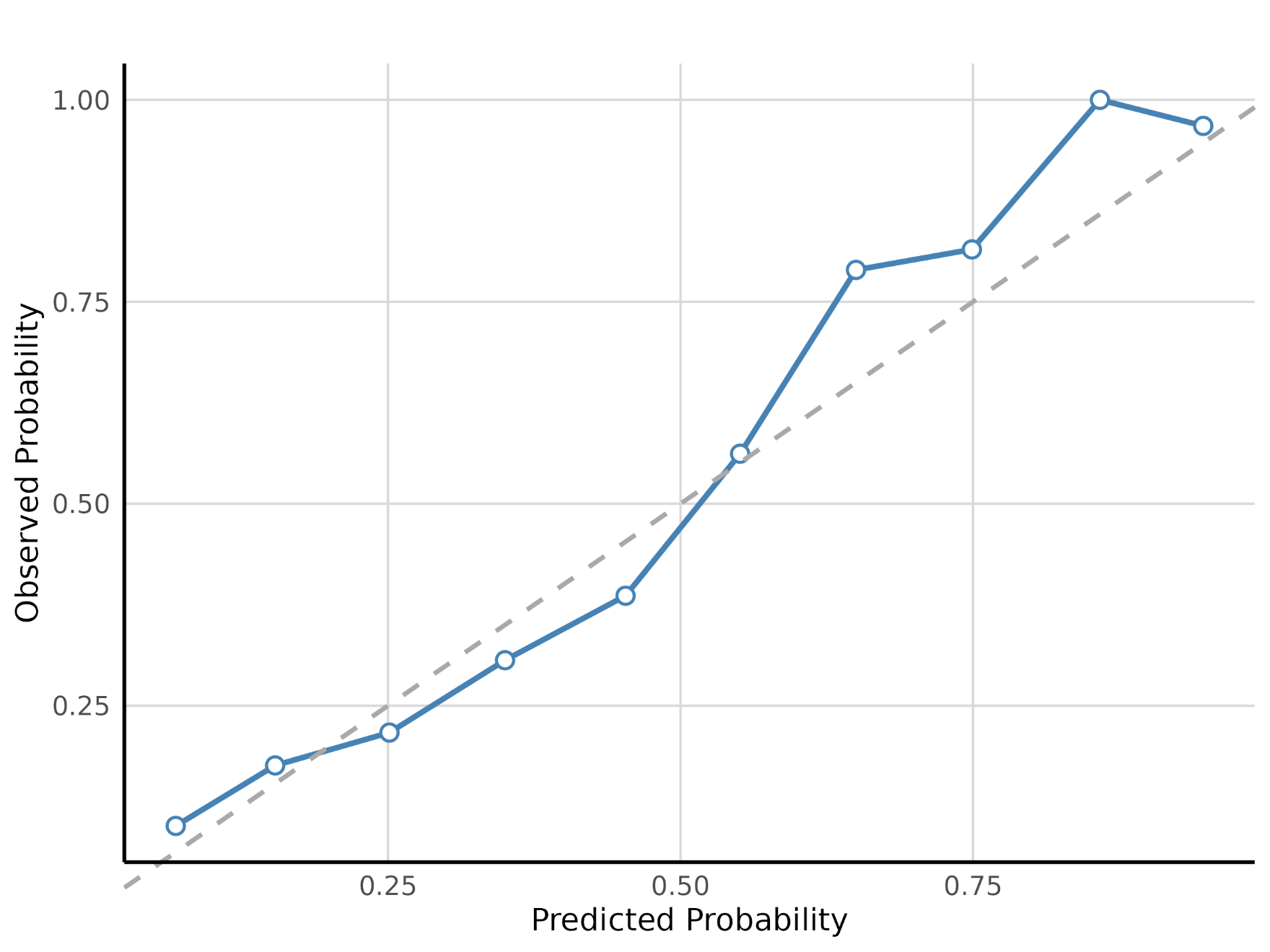
**

**Supplemental figure 2**. Dynamically updated predicted probabilities of lifetime AD risk based on cognitive assessment measurements for two selected participants: participant A, who remained cognitively intact throughout the follow-up period, and participant B, who developed incident AD. In the upper graphs, cognitive function scores over several years were shown for each participant. Participant A’s cognitive functions remained relatively stable or exhibited slight variability without a clear downward trend. In contrast, participant B showed more pronounced fluctuations and an overall decline in cognitive scores, particularly in global cognitive function and episodic memory. The lower graphs illustrated the corresponding predicted probabilities of lifetime AD risk, including 95% prediction intervals. For participant A, the AD risk remains consistently low, with narrow prediction intervals indicating high confidence in the assessment. Conversely, participant B experienced a significant increase in predicted AD risk from the third year onward, which aligns with the observed decline in their cognitive scores.

**
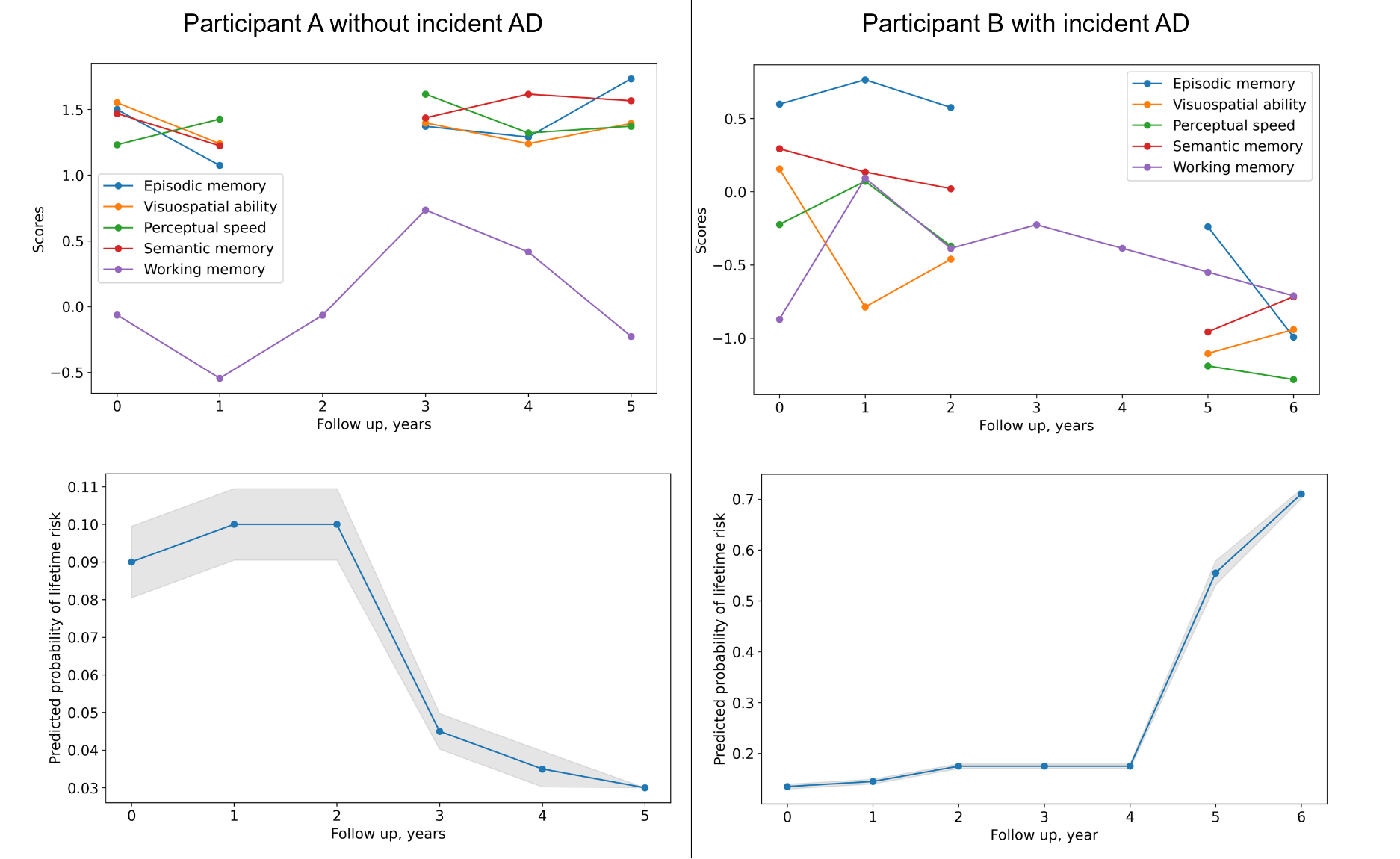
**
